# Time-Resolved Thickness and Shape-Change Quantification using a Dual-Band Nanoplasmonic Ruler with Sub-Nanometer Resolution

**DOI:** 10.1021/acsnano.2c04948

**Published:** 2022-09-09

**Authors:** Ferry Anggoro Ardy Nugroho, Dominika Świtlik, Antonius Armanious, Padraic O’Reilly, Iwan Darmadi, Sara Nilsson, Vladimir P. Zhdanov, Fredrik Höök, Tomasz J. Antosiewicz, Christoph Langhammer

**Affiliations:** † Department of Physics, Chalmers University of Technology, 412 96 Göteborg, Sweden; ‡ Department of Physics and Astronomy, Vrije Universiteit Amsterdam, De Boelelaan 1081, 1081 HV Amsterdam, The Netherlands; § Department of Physics, Universitas Indonesia, Depok 16424, Indonesia; ^ Faculty of Physics, University of Warsaw, Pasteura 5, 02-093 Warsaw, Poland; ∥ Department of Health Sciences and Technology, ETH Zurich, 8092 Zurich, Switzerland; ∇ Boreskov Institute of Catalysis, Russian Academy of Sciences, Novosibirsk 630090, Russia

**Keywords:** biomolecules, biosensors, conformation, nanoplasmonic sensors, nanorulers, supported lipid bilayer

## Abstract

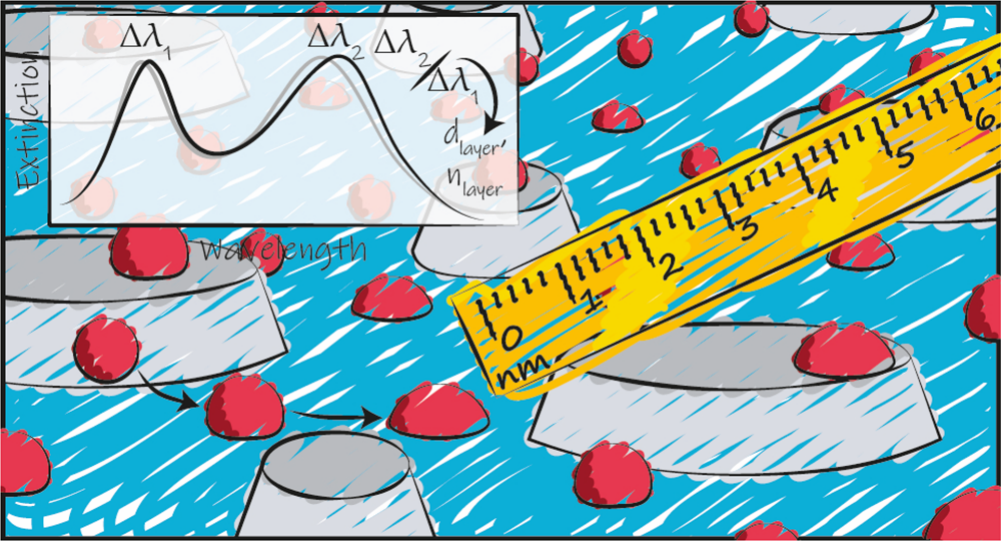

Time-resolved measurements of changes in the size and shape of nanobiological objects and layers are crucial to understand their properties and optimize their performance. Optical sensing is particularly attractive with high throughput and sensitivity, and label-free operation. However, most state-of-the-art solutions require intricate modeling or multiparameter measurements to disentangle conformational or thickness changes of biomolecular layers from complex interfacial refractive index variations. Here, we present a dual-band nanoplasmonic ruler comprising mixed arrays of plasmonic nanoparticles with spectrally separated resonance peaks. As electrodynamic simulations and model experiments show, the ruler enables real-time simultaneous measurements of thickness and refractive index variations in uniform and heterogeneous layers with sub-nanometer resolution. Additionally, nanostructure shape changes can be tracked, as demonstrated by quantifying the degree of lipid vesicle deformation at the critical coverage prior to rupture and supported lipid bilayer formation. In a broader context, the presented nanofabrication approach constitutes a generic route for multimodal nanoplasmonic optical sensing.

The ability to accurately measure the size and shape of nanoscale objects is one of the key achievements of nanoscience and nanotechnology, since these characteristics dictate many properties and functionalities of nanoscale objects in materials and life science.^1−4^ In the latter area, precise characterization of nanoscopic biological entities is especially important, e.g., for the development of advanced diagnostics and therapeutic systems and tools, since many diseases are induced by a modified functionality of such entities due to changes in their structure or conformation. For example, protein misfolding and amyloid fibril formation is associated with Alzheimer’s disease^5^ and an increased size of saliva and urinary exosomes is correlated with the occurrence of oral^6^ and prostate cancers,^7^ respectively. On the other hand, the size of lipid nanoparticles and exosomes is also a critical parameter for their efficiency in drug delivery.^8,9^ At the same time, performing an accurate size and shape determination of nanoscopic biological entities is challenging because they are “soft” and highly dynamic and because their dimensions and conformation depend on interactions with other biological species or surfaces in their surroundings. To this end, various techniques have been used to quantify such systems, including transmission electron microscopy (TEM),^10−12^ X-ray crystallography,^13,14^ neutron reflectometry,^15,16^ and nuclear magnetic resonance (NMR).^17,18^ However, TEM seldomly enables characterization of the dynamics of such processes and thus prohibits studies of conformational change in real time.^19,20^ This situation is similar to X-ray crystallography due to its requirement of crystalline samples. In contrast, both neutron reflectometry and NMR permit the study of conformational dynamics, however, only with low throughput and high sample consumption.^15−18^

In this regard, optical sensing techniques, such as ellipsometry,^21^ silicon microring resonators (SMR),^22,23^ optical waveguide lightmode spectroscopy (OWLS),^24,25^ and surface plasmon resonance (SPR),^26−28^ are attractive tools since they provide sensitive, label-free, and real-time detection with high throughput and relatively simple instrumentation. Conceptually, these methods all measure the presence of, or a change in, a biomolecular layer formed on the sensor surface via changes in the interfacial refractive index. Due to a near-linear correspondence between changes in this index and the number of bound molecules, these methods are commonly used to measure the adsorbed molecular mass with high accuracy. Furthermore, by employing intricate modeling and multiparameter measurements, both the refractive index and thickness of the biomolecular layer can be determined using these methods. Specifically, with state-of-the-art instrumentation and careful calibration of the substrate and solutions, ellipsometry, SMR, and OWLS can be used to quantify dense biomolecular layers with thicknesses down to 2 nm,^22,23,29,30^ and using multimode optical excitation concepts, similar deconvolutions are possible in the case of SPR.^22,23,25,31,32^

In this respect, the dual-mode SPR approach introduced by Rupert et al.^33^ for quantification of nanoparticle size and structure is of particular relevance for our work. They utilized and extended a formalism derived from the characteristic response of an SPR sensor^32,34^ that relates the ratio of the wavelength-shift response of the two considered SPR modes to the size, shape, and RI of the studied systems.^33^ However, due to the large extent of the evanescent field from the surface in SPR (100–400 nm), this method is only fairly accurate in the quantification of the mass of nanoparticles on this or smaller length scale (e.g. tens of nanometers), and thus it is, among others, not capable of characterizing the (change of) shape of nanoparticles with sizes smaller than few tens of nanometers.

In contrast, localized surface plasmon resonance (LSPR) based sensors hold promise for the characterization of analytes of few tens of nanometers and smaller due to the significantly shorter field decay lengths^35^ and have been successfully employed to determine distances of few nanometers, e.g., among two plasmonic antennas and a mirror between and an antenna and a biological layer.^36−39^ Furthermore, they have been used to scrutinize changes in the shape of adsorbed biomolecules^40−42^ and biological nanoparticles, such as lipid vesicles under various conditions,^43−47^ and have been employed to investigate the remodeling of a supported lipid bilayer with the formation of buds and tubules.^48^ In such studies, the LSPR sensors can be viewed as a nanoplasmonic ruler that enables thickness measurements. To this end, in a very recent attempt to combine SPR and LSPR, Mataji-Kojouri et al. have developed a Fabry–Perot cavity array that supports these modes in the same structure. Although this solution performs better than SPR nanorulers in that it is able to determine the thickness of a biomolecular layer in the range of 10–100 nm, the obtained resolution is limited to 4 nm.^31^

To push the plasmonic nanoruler concept to the regime where an accurate layer thickness determination becomes possible with sub-nanometer resolution, we introduce here a dual-band nanoplasmonic ruler comprising two mixed populations of plasmonic nanoantennas with distinctly different sizes that gives rise to two independent LSPR peaks in the extinction spectrum. Due to the short-range evanescent fields created by the resonating antennas, this approach enables real-time and accurate thickness and RI determination in the sub 10 nm layer thickness range. To corroborate this dual-band nanoplasmonic ruler concept theoretically, we first thoroughly assess the—*a priori* not obvious—applicability of the formalism introduced by Rupert et al.^33^ for SPR sensors to LSPR sensors using electrodynamic simulations based on the finite-difference time-domain (FDTD) method and use it to rationally design the LSPR ruler that we subsequently implement in practice. For that purpose, we employ a tailored version of hole-mask colloidal lithography and demonstrate the ability of the nanofabricated dual-band nanoplasmonic ruler to accurately measure and temporally resolve the thickness change in different systems and settings: (i) atomic layer deposition of an Al_2_O_3_ film in air, (ii) adsorption of 35 nm lipid vesicles with subsequent spontaneous formation of a planar supported lipid bilayer (SLB) on silica in liquid, (iii) adsorption of 7 nm SiO_2_ nanospheres onto an SLB, and (iv) quantification of the shape changes of adsorbed lipid vesicles during SLB formation, demonstrating sub-nanometer resolution up to a thickness accumulation/change of 60 nm, which constitutes a 1 order of magnitude higher resolution than state-of-the-art SPR nanorulers.

## Results and Discussion

### Theoretical Background

In conventional SPR, the response (or, more specifically, the shift in either wavelength or angle of the SPR minimum) associated with changes in the RI and/or thickness and shape of a nanosized analyte (referred to below as “layer”) in contact with the metal surface of the sensor can be analytically described as (see eq 6 in combination with eq 4 in ref (33))
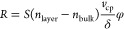
1where *S* is the sensor sensitivity, *n*_bulk_ and *n*_layer_ are the bulk and layer RIs, respectively, *v*_cp_ is the molecular analyte volume per unit area (in fact, this is the average thickness of the analyte layer, i.e., the thickness calculated with the close-packed arrangement of analyte atoms or molecules), φ (≤1, or 1 provided the layer is thin and the field extinction is negligible) is a dimensionless factor taking the decay of the intensity of the evanescent field into account that is defined (eq 7 in ref (33)) as a normalized convolution of the analyte-mass distribution with the exponential attenuation function exp(−*z*/δ), and δ is the corresponding decay length. This expression for *R* depends on the analyte optical properties via *n*_layer_ and geometry via φ, and, accordingly, their values cannot be inferred simultaneously from measurements at a single frequency. With two frequencies (associated below with subscripts 1 and 2), however, this is possible and it is convenient to use the ratio of the two readouts (eq 15 in ref (33))
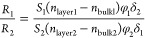
2

In applications, the values of all the parameters (except φ_1_ and φ_2_) are either known or their ratio can be determined by performing measurements in the thin-layer limit (with φ_1_ = φ_2_ = 1). Then eq 2 can be applied to the layers with an arbitrary fine structure and size, and these properties can be characterized via the ratio φ_1_/φ_2_. We are interested below primarily in a uniform close-packed layer of thickness *d*_layer_ with *v*_cp_ = *d*_layer_ (eq 14 in ref (33)) and
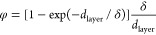
3

In this case, eqs 1 and 2 can, respectively, be rewritten as
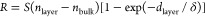
4and
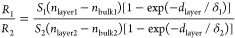
5

We are now interested to investigate in which way this SPR formalism can be applied to an LSPR sensor. Here we note, that despite the obvious conceptual similarities between SPR- and LSPR-type sensors, it is not *a priori* clear that the above formalism derived for SPR can be validly extended to LSPR, with the distinctly different decay lengths, δ, for SPR (100–400 nm) and LSPR (few tenths of nanometers) and the markedly different geometrical factors (extended planar vs point/localized-like structure) as the main reasons. Thus, a rigorous analysis using electrodynamic simulations that we do here below is an imperative first step to validate the subsequent development of a dual-band nanoplasmonic ruler. For this purpose, we first recall that, in SPR, the decay of the evanescent field is exponential and the corresponding decay length, δ, is defined by the light frequency and optical constants of the media. In LSPR, in contrast, the evanescent field around sensing nanoantennas contains different terms (dipole, etc.), and the corresponding decay length is roughly proportional to and significantly smaller than the antenna size. In addition, one should operate by the permittivities rather than by RIs. At the simplest level this difference can be taken into account in the dipole approximation by just replacing the exponential attenuation function, exp(−*z*/δ), in the calculation of φ by the power-law function, ∼1/(R* + *z*)^6^, where R* is the length scale (effective radius) characterizing plasmonic nanoantennas.^46^ In the context of applications, the difference between these two approaches is often minor.^47^ For this reason, we use here the conventional exponential approximation, or more specifically eqs 4 and 5, in the LSPR case in order to articulate the analogy between SPR and LSPR, as has often been done in earlier literature since the first applications of LSPR (see, e.g. ref (49)). In particular, we identify *S* with the bulk refractive index sensitivity, BRIS (to be distinguished from the local sensitivity of an LSPR sensor), and rewrite eq 5 as
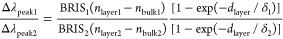
6where Δλ_peak*i*_ (*i* = 1, 2) are the analyte-induced LSPR peak position shifts of two sensing plasmonic nanoantennas. Since both resonances practically measure the same analyte layer in the same medium and in our context the dependence of *n*_layer_ and *n*_bulk_ on the light frequency is negligible,^50^ i.e., *n*_layer 1_ = *n*_layer 2_, and *n*_bulk 1_ = *n*_bulk 2_, the expression can be further simplified to
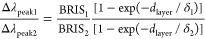
7

This equation then contains only one unknown: the *d*_layer_ of the analyte layer, which can be conveniently derived, given that BRIS_*i*_ and δ_*i*_ have been previously determined in calibration experiments of the sensor. Equation 7 forms the basis for our analysis below.

In the LSPR case, as already noted, eqs 6 and 7 correspond to the simplest phenomenological approximation containing one length scale, δ. This approximation is not exact even in the case of a uniform close-packed layer, because the evanescent field around sensing nanoantennas is inherently not exponential (it depends on their shape and is influenced by the support). Nevertheless, the usefulness of eqs 6 and 7 in the LSPR context has already been illustrated.^49,51−53^

To explicitly clarify this aspect in our case, we employ finite-difference time-domain (FDTD) simulations to model LSPR sensors comprising Au nanodisks, with diameters spanning from 60 to 180 nm and thicknesses from 20 to 70 nm. Au (rather than Ag that we later use in our experimentally implemented LSPR nanoruler) is chosen, since it is by far the most commonly used SPR surface (which also was used in ref (33), which forms the basis for our analysis here). The insights obtained from our simulations on Au can be directly translated to other noble metals, such as Ag that we use below, since both metals have a similar plasma frequency of around 9 eV and beyond the interband range (which is the one of interest here, especially due to the red shift caused by the substrate and water) their permittivies are similar.

**Figure 1:**
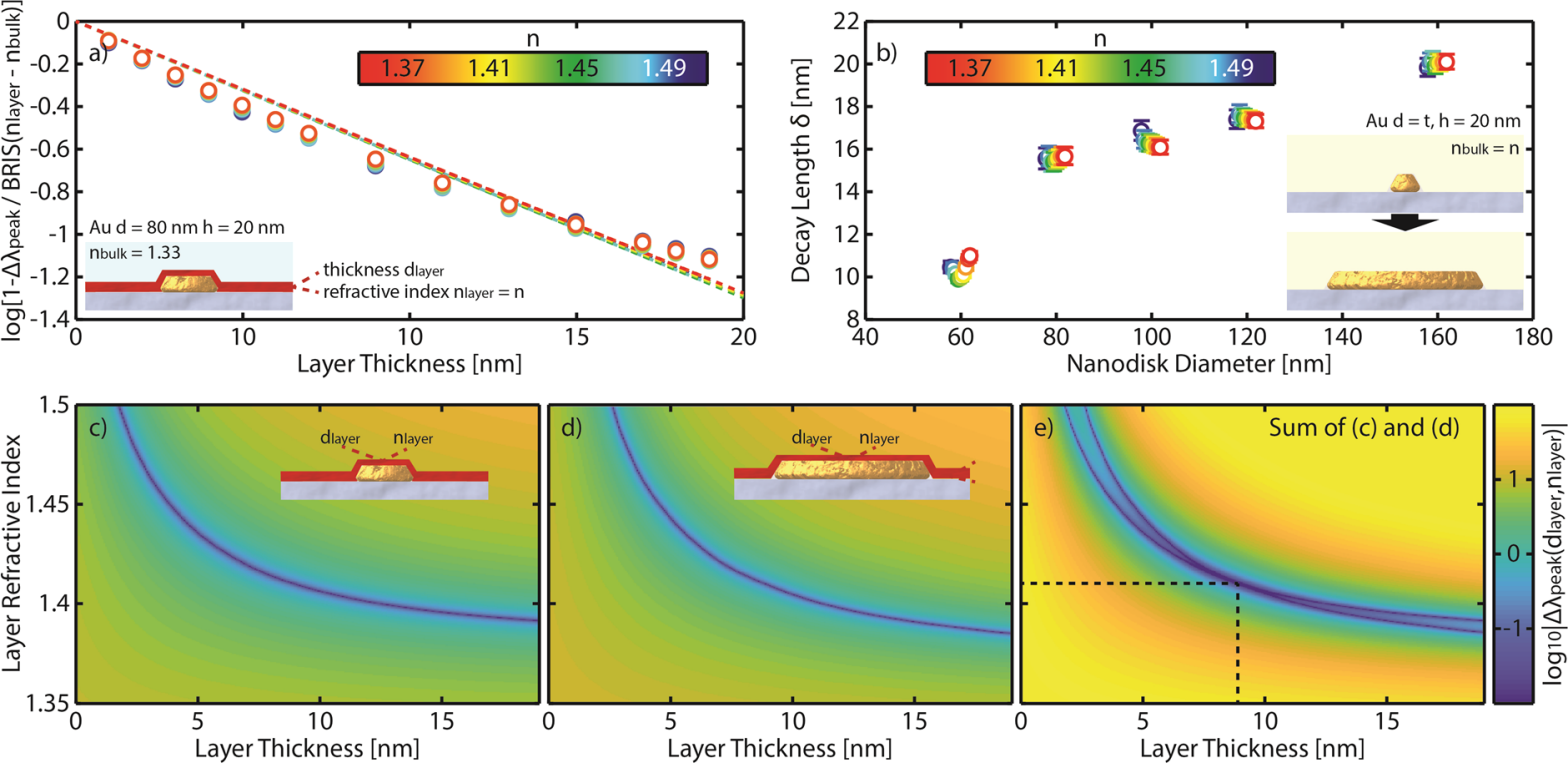
Design consideration for a dual-band nanoplasmonic ruler. (a) FDTD-calculated Δλ_peak_ of a Au nanodisk in water covered with thin conformal layers of up to 20 nm thickness and with RI of 1.35–1.5, expressed as eq 8, as a function of the layer thickness (the data points are presented by using BRIS calculated in Figure S1, which is used as a fitting parameter). The dashed lines are the exponential approximation of the LSPR sensitivity, in which the linearity confirms that the SPR model is a good approximation for the inhomogeneous fields in a nanodisk, whose effective decay length, δ, is given by the inverse slope of the dashed lines. The inset shows a to-scale schematic of the simulated system. (b) The effective decay lengths, δ, for nanodisks of 20 nm height and varying diameters in media of different RIs. Error bars represent the confidence intervals of the linear regression in fitting the logarithm of the exponential function in eq 8. The data are offset in the *x* axis for clarity. (c, d) Δλ_peak_ of a nanoplasmonic sensor comprising a Au nanodisk of height 20 nm and (c) 80 nm and (d) 160 nm diameter, coated with a conformal dielectric layer with various thicknesses, *d*_layer_, and RIs, *n*_layer_, subtracted by Δλ_peak_ of the corresponding sensors when *d*_layer_ and *n*_layer_ are 9 nm and 1.41, respectively. It is clear that, knowing only Δλ_peak_, it is impossible to deduce *d*_layer_ and *n*_layer_, as there are infinite combination possibilities that result in a similar Δλ_peak_ compared to when the surface is covered with a layer of 9 nm with an RI of 1.41. (e) Combining the two independent results of (c) and (d) enables an unambiguous determination of both *d*_layer_ and *n*_layer_, i.e., 9 nm and 1.41, respectively (dashed lines).

In our simulations, the Au nanodisks are placed on a substrate with a RI of 1.5 and either they are surrounded by a medium with RIs ranging from 1.33 to 1.5 to emulate a BRIS experiment or they are covered by a thin conformal layer of up to 20 nm thickness with an RI of up to 1.5 to emulate a molecular/thin-layer sensing experiment. For the first scenario, the BRIS is a linear function of the nanodisk dimensions and scales well across the simulated parameter range, as expected (Figure S1). To showcase the key findings for the thin-layer sensing scenario, we use a nanodisk with a diameter of 80 nm and a thickness of 20 nm as an example. However, instead of directly plotting the obtained peak shift Δλ_peak_ vs *d*_layer_ for different RIs of the layer, we modify the former into the form
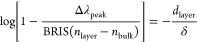
8which is derived from eq 4 with appropriate parameters for an LSPR sensor (i.e., Δλ_peak_ and BRIS replacing *R* and *S*, respectively). The fit of our FDTD calculations by using the left-hand part of eq 8 is close to linear, as predicted by eq 4 (Figure 1a). Thus, eqs 4 and 7 indeed describe our system in an acceptable way. In addition, our calculations show that, as expected,^46,54^ (i) the dependence of δ on RIs is very weak and thus can be neglected (Figure 1b) and (ii) δ is significantly shorter than the size (diameter) of the sensing nanoantenna and increases with an increase in this size, because the decay of the evanescent field is determined primarily by the shape of the nanoantenna (in the dipole approximation,^46^ one has δ ≃ R*/5).

### Nanoruler Design Considerations

Having confirmed the overall validity of our formalism for LSPR-based sensing, we apply it to design a dual-band nanoplasmonic sensor surface that we subsequently implement and explore experimentally. In this process, the first design consideration is to choose two plasmonic nanodisk types with spectrally nonoverlapping LSPR modes, different decay lengths, and sufficient sensitivities. To illustrate this concept explicitly, we simulated nanodisks with 80 and 160 nm diameter and identical thicknesses of 20 nm and placed a conformal dielectric thin layer with arbitrarily chosen thickness *d*_layer_ = 9 nm and RI *n*_layer_ = 1.41 on top of them to calculate the induced Δλ_peak_ for both nanodisk sizes for this condition. While this is straightforward, if we subsequently want to reverse this process and back-calculate which combination of *d*_layer_ and *n*_layer_ gave rise to the obtained Δλ_peak_ values for each disk, it turns out to be essentially impossible since, as outlined above, a practically infinite combination of *d*_layer_ and *n*_layer_ will produce a similar Δλ_peak_. To illustrate this condition, we calculated all of these potential matches (*d*_layer_ from 0 to 20 nm and *n*_layer_ from 1.35 to 1.5) for the two disks independently (Figure 1c,d). Specifically, we plot the values of , in that the minima give the combination of *d*_layer_ and *n*_layer_ that results in a peak shift similar to . While separately the individual response of each of the two nanodisks is not unique with respect to *d*_layer_ and *n*_layer_, the sum of these two plots yields a single point at which the minima of the two curves intersect, which corresponds to the initially chosen values of *d*_layer_ = 9 nm and *n*_layer_*=* 1.41, respectively (Figure 1e). Mathematically, such a distinct intersection point can only be defined if the two disks have distinctly different sensitivities and decay lengths, since the particular dependences otherwise partially overlap rather than intersect each other. This, in turn, would result in a large uncertainty of the unique combination of layer thickness and RI that is compatible with the LSPR response of both disk types. Hence, to realize a dual-band nanoplasmonic ruler, one has to employ two types of plasmonic nanoantennas with not only spectrally well separated peaks but also with distinctly different sensitivities and field decay lengths.

We also note that slight disparities of nanodisk geometry due to process variations during fabrication will not affect the functionality of the sensors, as the two disk geometries are chosen with sufficient differences in their diameters to preserve their unique sensing characteristics even when they are subject to fabrication inaccuracies. At the same time, we note that such variations will result in sensors with slightly different sensitivities and decay lengths and therefore lead to slightly different peak ratio-to-thickness conversion plots for their calibration.

### Nanoruler Fabrication and Characterization

Conceptually, a nanoplasmonic ruler that can disentangle thickness and refractive index variations as outlined above can be accomplished by executing identical measurements on two separate plasmonic surfaces that feature spectrally separated resonance peaks. However, while such measurements are sufficient if one is interested in steady-state conditions, they cannot temporally resolve the investigated processes and therefore preclude analysis of, e.g., kinetics, since it is almost impossible to have exactly identical conditions in separate experiments on two different surfaces. Therefore, we develop a nanoplasmonic ruler surface where two nanoantennas of different types with distinctly different sizes and thus plasmon resonance wavelengths are mixed within the same array and thus on the same surface. In this way, only one experiment is required to quantify thickness, refractive index, and adsorbate shape, as well as temporal variations in the properties during dynamic processes.

To implement a dual-band nanoplasmonic ruler in line with this design principle, while simultaneously being compatible with the visible to near-infrared (NIR) wavelength range (400–1100 nm) most commonly used in the field,^55^ we identify two Ag nanodisk populations with 80 and 210 nm diameter and similar height of 20 nm as the best-suited nanoantennas for our purpose.^52,56^ Specifically, due to Ag’s narrow LSPR modes and interband absorption threshold of ∼325 nm, a distinct spectral separation of the LSPR peaks can be obtained.

**Figure 2:**
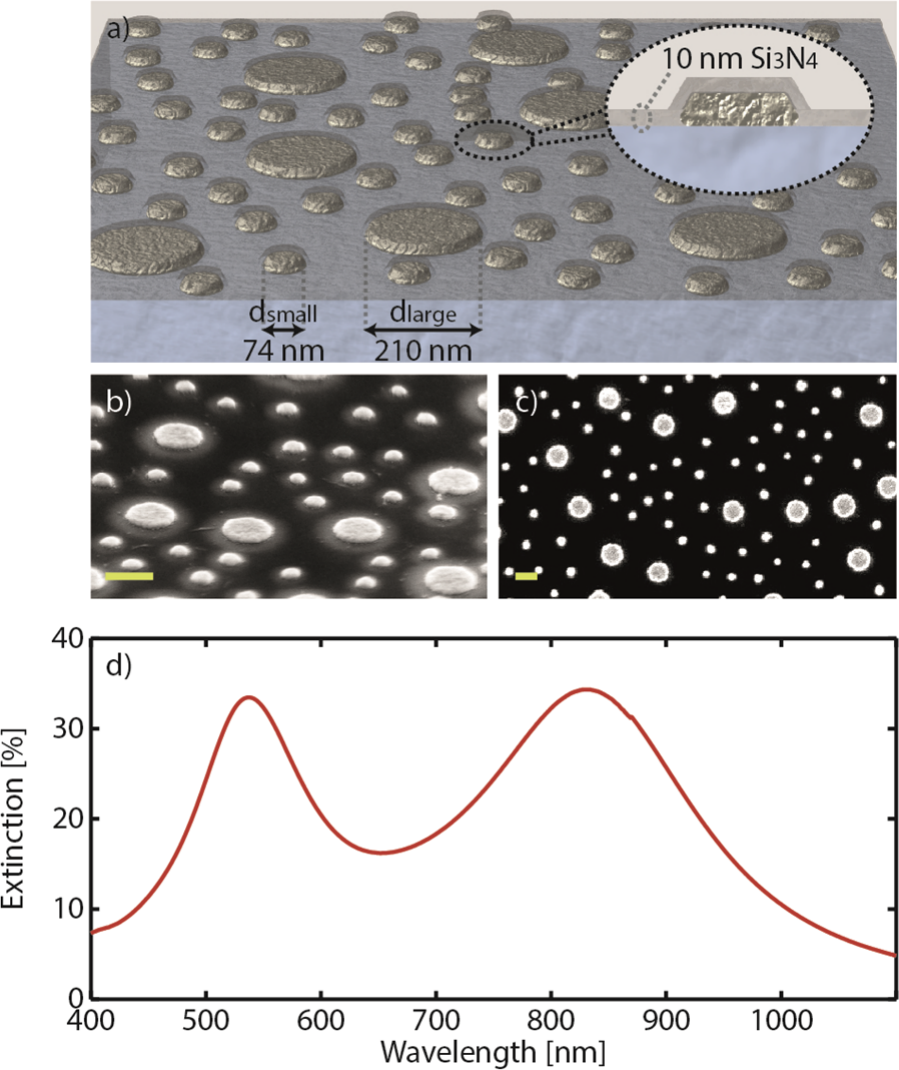
Dual-band nanoplasmonic ruler design and optical spectra. (a) Artist’s rendition of the sensor surface comprising a quasi-random array of two distinct populations of Ag nanodisks with diameters *d*_small_ = 74 nm and *d*_large_ = 210 nm, both with a height *h* = 20 nm, covered with a conformal 10 nm thin Si_3_N_4_ coating (inset). (b) Tilted and (c) normal incidence SEM images of the corresponding nanostructured surface. Note that the imaged sensor is not coated with Si_3_N_4_ for better image contrast. Scale bars are 200 nm. (d) Optical extinction spectra of the dual-band nanoplasmonic ruler sensor characterized by two spectrally distinctly separated peaks with identical intensities.

To implement these properties on a single surface, we employed a modified version of the hole-mask colloidal lithography (HCL)^57^ method, using a polystyrene (PS) colloidal suspension for the self-assembly step, in which PS beads of two distinctly different sizes (here 74 and 210 nm nominal diameter) were mixed to create a HCL-mask with uniformly distributed holes of two different diameters defined by the beads (see Methods for details). Using this approach, we were able to produce mixed arrays of two nanodisk types with dissimilar sizes on a substrate in a single HCL fabrication cycle (Figure 2a). Varying the relative concentration of the two types of beads in the mixed suspension offers a means to control the relative abundance of the two nanodisk sizes in the mixed array on the surface and thus the relative intensity of the respective LSPR peak, where the aim was to achieve roughly equal intensities (Figure S2). To this end, mixing 0.02 wt % of 74 nm PS beads and 0.1 wt % of 210 nm PS beads in water results in randomly mixed nanodisk arrays whose extinction spectra exhibit two distinguishable LSPR peaks of similar intensity and with a large spectral separation (Figure 2b–d and also see Figure S3 for the nanodisk size distribution). Finally, to introduce both long-lasting structural integrity and protection from harsh chemical and temperature conditions,^52,58−63^ as well as to provide a uniform chemical surface, we applied a thin conformal Si_3_N_4_ coating to the sensor surface (Figure 2a). If needed, other coating materials can be utilized (e.g. SiO_2_, TiO_2_) to alter the interaction of the adsorbed films/biomolecules with the support.

**Figure 3:**
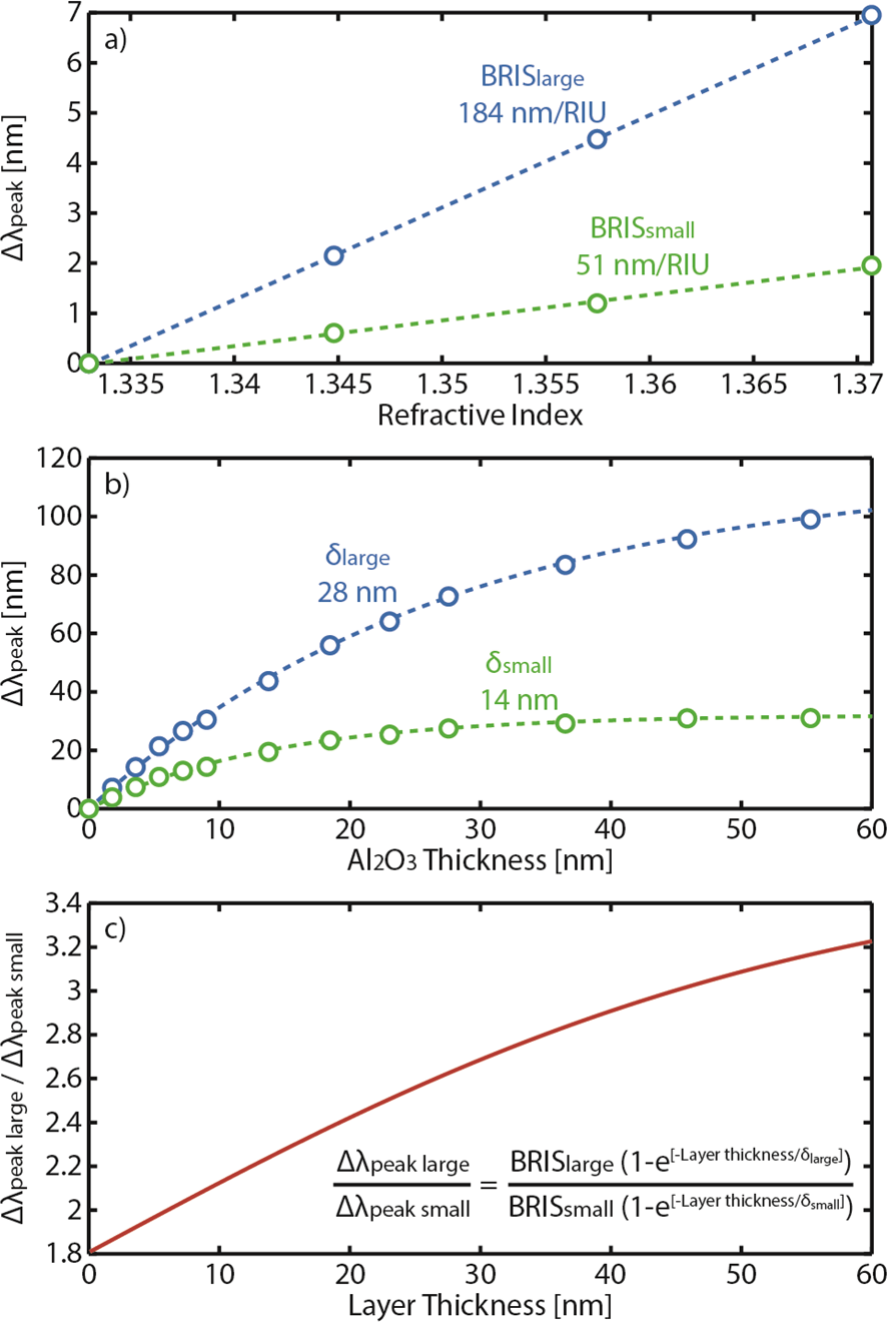
Sensitivity, field decay length determination, and sensor response-to-layer thickness conversion plot of the dual-band nanoplasmonic ruler. (a) Bulk refractive index sensitivity, BRIS, and (b) decay length, δ, determination for the small and large nanodisks in the mixed array. Dashed lines in (a) and (b) are linear fits and a fit of the data to eq 4,^34,52^ respectively. (c) Nanoruler conversion plot which translates the Δλ_peak_ ratio of the large and small nanodisks into the (cumulative) thickness of the layer on top of the sensor.

With the sensor surface at hand, we next experimentally quantify the two key sensitivity descriptors of the two types of nanodisks in the array, namely their BRIS and field decay lengths δ. The BRIS is derived by correlating the shifts of the two LSPR peaks, Δλ_peak_, to the RI of the medium the sensor is exposed to (Figure 3a; see Figure S4 for raw data). It is clear that the two nanodisk populations in the mixed array respond independently and exhibit different BRIS, i.e., BRIS_small_ = 51 nm/RIU and BRIS_large_ = 184 nm/RIU. These sensitivities are consistent with previous experimental and theoretical works demonstrating a positive correlation between a plasmonic particle size and BRIS.^64,65^

To determine the field decay lengths of the two types of nanodisks in the mixed array, we applied the established method of subsequent atomic layer deposition of thin Al_2_O_3_ layers and fitting the resulting Δλ_peak_ induced by the thickness accumulation of each layer to the local sensitivity of a plasmonic nanoantenna expressed in eq 4.^34,52,61,62,66^ As depicted in Figure 3b, deposition of conformal Al_2_O_3_ thin layers of up to 55 nm gives rise to increasing and eventually saturating Δλ_peak_ for both small and large nanodisks. To this end, the small nanodisks reach Δλ_peak_ saturation earlier, around a layer thickness of 40 nm, implying insensitivity to thickness change beyond this value. The large nanodisks, on the other hand, still exhibit a discernible Δλ_peak_ even beyond a layer thickness of 55 nm due to their anticipated longer field decay length.^52,54^ To explicitly extract the decay length from our data, we fit the Δλ_peak_ response to eq 4 and find δ_small_ = 14 nm and δ_l__arge_ = 28 nm. Notably, these values compare well to those of the Au nanodisks simulated above (cf. Figure 1b).

After this analysis we have all the necessary input to construct the nanoruler conversion correlation given by eq 7 for our nanofabricated sensor (Figure 3c). The obtained conversion plot provides a direct translation between the Δλ_peak_ ratio between the large and small nanodisks obtained from a measurement and the corresponding thickness of an arbitrary layer deposited on the sensor. This conversion plot is strictly valid only for experiments conducted in an air/gaseous environment. In other words, if the sensor surface is used for a measurement in other media, e.g. water, a new conversion plot needs to be constructed on the basis of sensitivity parameters (in particular the decay lengths) determined in this medium. However, as we explicitly show below, by using a system with well-known thickness and RI in liquid medium, we can simplify the steps for finding the decay lengths of the nanodisks. Furthermore, now with the two sensitivity descriptors of the nanodisks known, modification of eq 4 also enables the nanoruler to determine the RI of the deposited layer, i.e.,
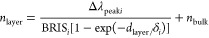
9

**Figure 4:**
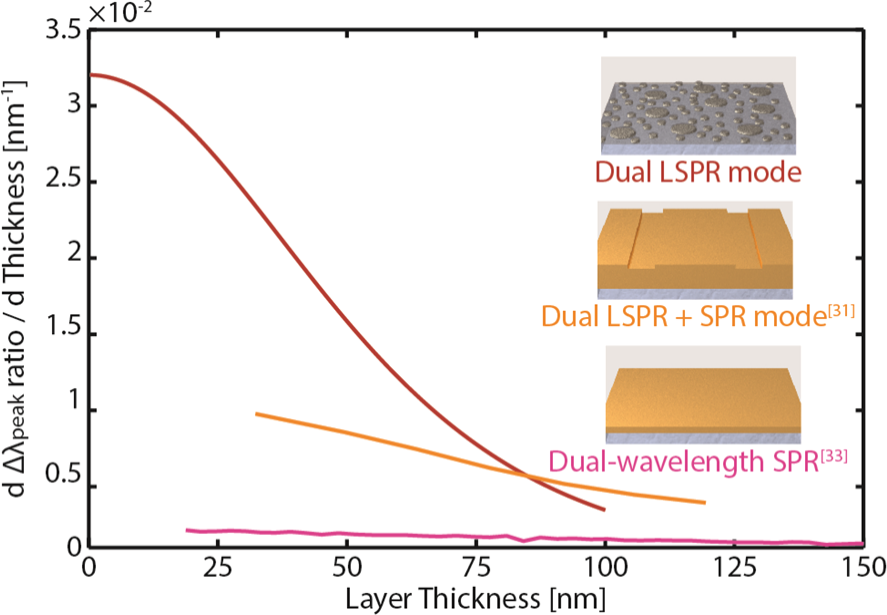
Dual-band plasmonic nanoruler sensitivity. The derivative of the conversion plot (cf. Figure 3c) reveals the sensitivity of the nanoruler, i.e., the change in the absolute Δλ_peak_ ratio per 1 nm change of the layer thickness. The triple sensitivity at a layer thickness up to ∼20 nm compared to those beyond ∼50 nm stems from the characteristic high local sensitivity of LSPR sensors, which is a consequence of the rapidly decaying near fields. The overall sensitivity of our dual-mode LSPR nanoruler in the few tens of nanometers thickness range is therefore at least twice as high as and 1 order of magnitude higher that those of the LSPR-SPR mode^31^ and dual-SPR mode^33^ solutions, respectively.

Finally, plotting the first derivative of the Δλ_peak_ ratio with respect to the layer thickness allows us to also assess the sensitivity of such nanorulers in terms of the absolute Δλ_peak_ ratio change per 1 nm change of the layer thickness. As shown in Figure 4, our system exhibits a corresponding sensitivity of ∼0.03 Δλ_peak_ ratio change for a layer thickness up to ∼20 nm, which then gradually decreases to about one-third of this value at a layer thickness of 60 nm. As a key point here, we highlight that the range within which the nanoruler exhibits the highest sensitivity toward a thickness change coincides well with the near-field decay length of the plasmonic nanodisks, which is on the order of 20 nm. This is obvious when we compare the sensitivity of our nanorulers with others employing LSPR-SPR modes^31^ and dual-SPR modes,^33^ whose sensitivities are at least 1/2 and 1 order of magnitude lower, respectively, due to the much longer decay lengths, which are on the order of few hundred nanometers in the SPR case. As the key conclusion, this comparison thus highlights that our LSPR-based dual-band nanoruler is most suitable for measurements of layers in the few to a few tenths of nanometers thickness range.

### Thickness and Refractive Index Determination of Single-Layer Accumulation in Air

**Figure 5:**
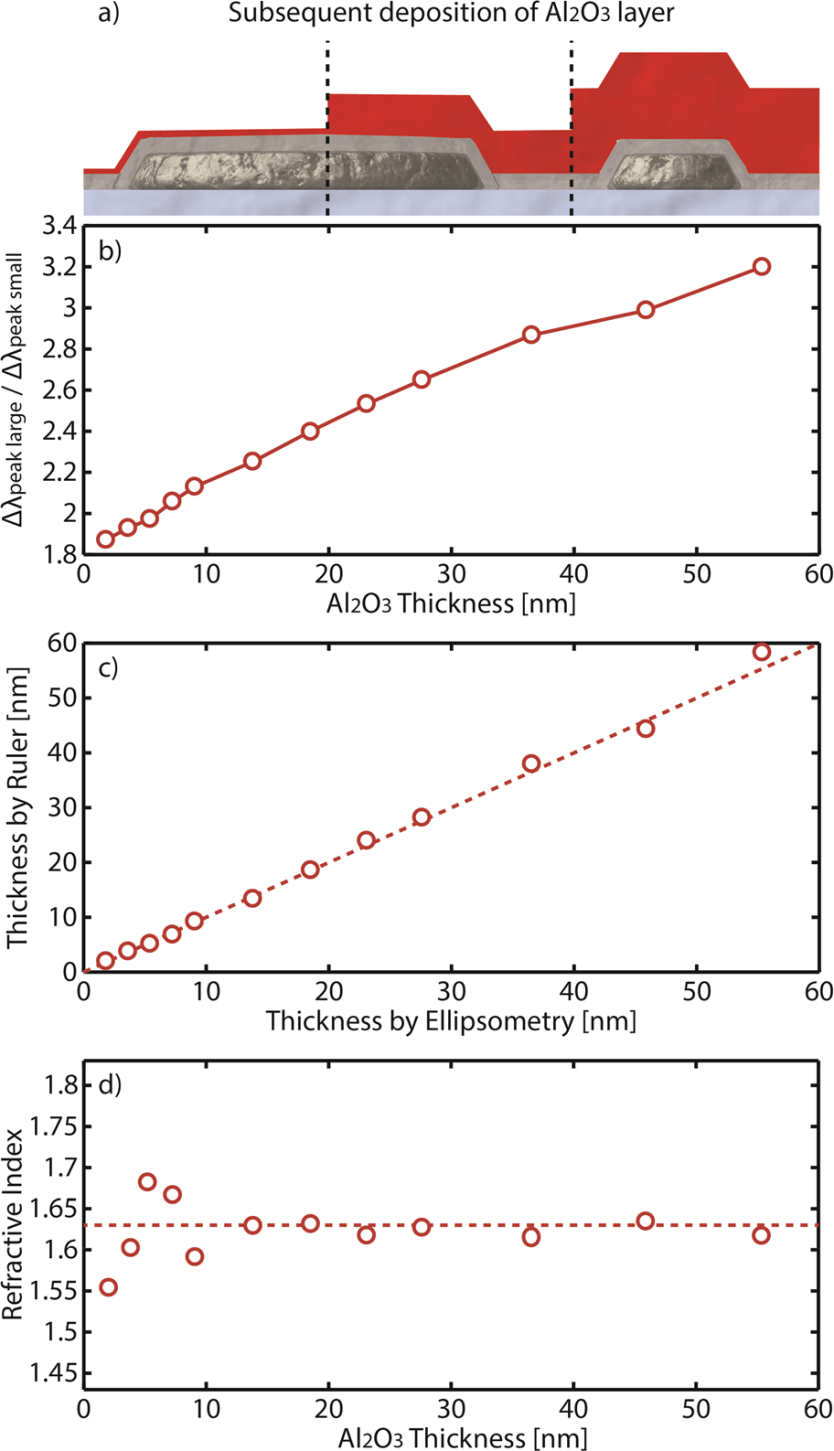
Measuring cumulative layer thickness and RI in air. (a) Schematic of the studied system comprising a gradual buildup of thin Al_2_O_3_ layers on top of a Si_3_N_4_-coated sensor. (b) Corresponding Δλ_peak_ ratios of the large and small nanodisks. (c) Comparison between the layer thicknesses derived with the nanoruler (i.e., by translating the Δλ_peak_ ratio to layer thickness through the conversion plot in Figure 3c) and using ellipsometry. The thickness determination by the nanoruler up to 60 nm is in excellent agreement with the values obtained by ellipsometry. The dashed line is the 1:1 relation between the abscissa and the ordinate. (d) Corresponding RI determination of the accumulated thin Al_2_O_3_ layers. The dashed line is the corresponding RI measured by ellipsometry.

With the overall concept and sensor surface established, we now apply it to measure the thickness of a single layer accumulated on the ruler surface. To do this, we can again resort to Al_2_O_3_ layer deposition, as used above for determining the decay lengths in air (Figure 5a). Starting from the independent Δλ_peak_ determined for both small and large nanodisks upon deposition of subsequent Al_2_O_3_ layers (cf. Figure 3b), we can construct their Δλ_peak_ ratio (Figure 5b) and, for each of the Δλ_peak_ ratios obtained after addition of a new Al_2_O_3_ layer, calculate a corresponding Al_2_O_3_ layer thickness by using the conversion (Figure 3c). Comparing the layer thicknesses obtained in this way by the nanoruler with values obtained by ellipsometry for identical Al_2_O_3_ layers reveals an excellent agreement for the whole measured range from 2 to 55 nm (Figure 5c). Particularly noteworthy is that the nanoruler is able to distinctly resolve the layer thickness in the sub 10 nm thicknesses regime with a maximum absolute thickness difference between the nanoruler and ellipsometry of only 0.6 nm—a significant improvement in accuracy compared to other nanorulers.^31^ Furthermore, as discussed above, the nanoruler is also capable of measuring the RI of the Al_2_O_3_ layer, which again is in good agreement with the value obtained by ellipsometry (Figure 5d).

### Thickness and Refractive Index Determination of Biomolecular and SiO_2_ Nanosphere Layer Accumulation in Liquid Medium

**Figure 6:**
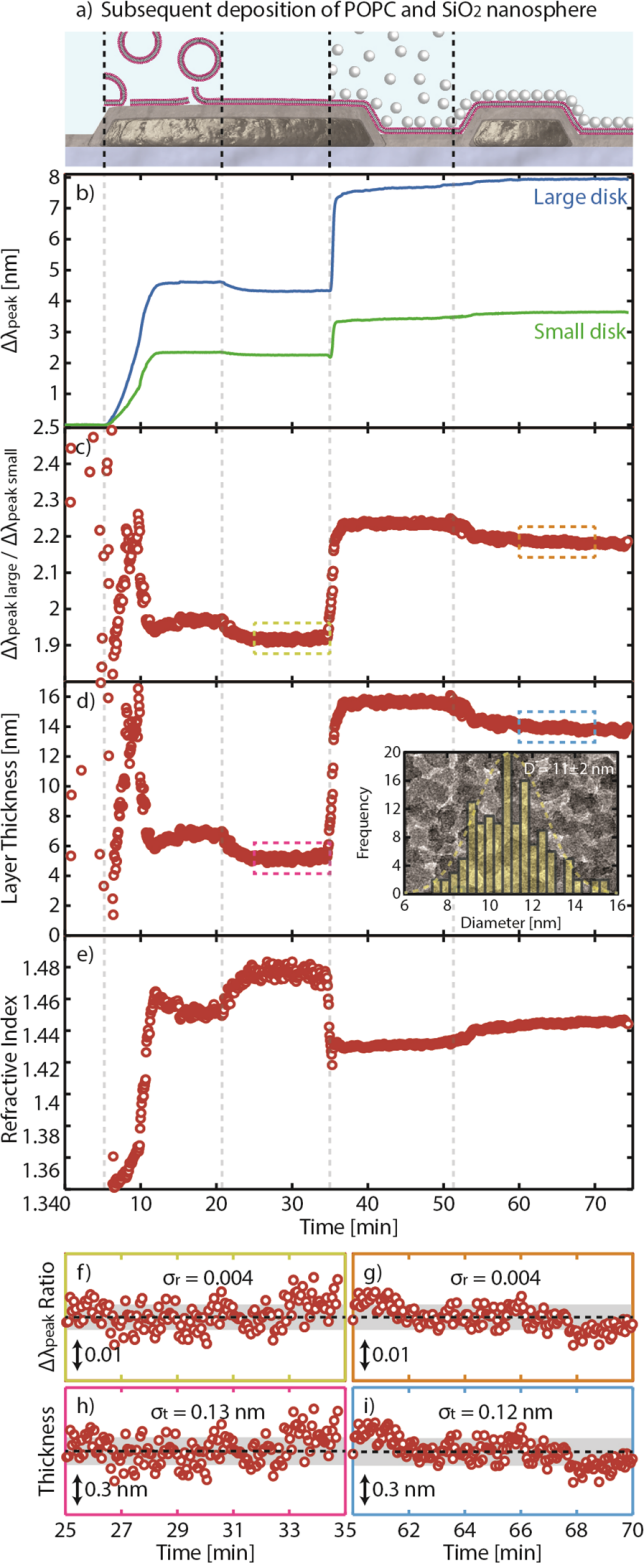
Measuring two different layers in liquid medium. (a) Schematic of the studied system and its various deposition phases: starting from a Si_3_N_4_-coated sensor, we deposit POPC vesicles that eventually rupture and form a supported lipid bilayer (SLB), onto which we adsorb SiO_2_ nanospheres that form a monolayer after rinsing. Note that the vesicles, SLB, and SiO_2_ nanospheres are drawn to scale to the Au nanodisk dimensions, while the binding conformations are arbitrary and should only serve as a schematic conceptual illustration. (b) Corresponding temporal evolution of the Δλ_peak_ of small and large nanodisks, showing markedly different responses as the deposition phase progresses, as delineated by the dashed lines. (c) Δλ_peak_ ratios of the small and large nanodisks. (d) The real-time thickness of the layer(s) determined by the nanoruler, showing the drastic thickness change prior to the SLB formation and subsequent thickness increase after the addition of the SiO_2_ nanospheres. Inset: size distribution histogram of SiO_2_ nanospeheres derived via TEM image analysis, with a representative bright-field TEM image in the background. The image is 120 × 80 nm^2^. (e) RI of the real-time layer(s) determined by the nanoruler. (f, g) Enlarged versions of panels (c) and (d) showing the typical Δλ_peak_ ratio and derived thickness, respectively, at two different occurrences in the experiment. Assessing the noise, it is clear that it is constant throughout the experiment and is small: i.e., σ_r_ = 0.004 and σ_t_ = 0.13 nm for the Δλ_peak_ ratio and thickness determination, respectively. Defining the limit of detection as 3σ_t_, the nanoruler is able to distinguish a 0.5 nm thickness difference for a total layer thickness up to 60 nm (cf. Figure 3c).

As a second demonstration of the dual-band LSPR nanoruler, we use it to characterize the multistep deposition of a supported lipid bilayer (SLB), followed by small SiO_2_ nanosphere adsorption (Figure 6a). Specifically, we first expose the nanoruler sensor to a lipid vesicle suspension (1-palmitoyl-2-oleyl-*sn*-glycero-3-phosphocholine, POPC) in BIS TRIS buffer, while continuously tracking the Δλ_peak_ of the two disk populations. Once saturation of the Δλ_peak_ is reached, which together with the observed characteristic “kink” in the plasmonic sensor signal (at ca. minute 10) imply that the POPC vesicles have ruptured and formed an SLB on the nanoruler,^67^ a rinsing step with pure buffer is applied to remove excess vesicles. Subsequently, we expose the nanoruler to a suspension of 7 nm SiO_2_ nanospheres, which physisorb onto the SLB. At the end, once Δλ_peak_ saturation has again been reached, a pure buffer rinse is applied to remove unbound spheres, leaving a monolayer of SiO_2_ nanospheres on the SLB. It is noteworthy that, in these experiments, the SLB represents a nearly perfectly uniform layer and accordingly can be characterized by using the nanoruler under consideration. In contrast, SiO_2_ nanospheres represent a heterogeneous layer, and it is expected to be characterized by the nanoruler with the simplest expression (eqs 6 and 7) only provided the nanosphere size is smaller than or comparable to δ that corresponds to the smaller sensing nanodisks. The size of the SiO_2_ nanospheres chosen for our experiment satisfies this condition.

To start the quantitative analysis and discussion of this experiment, we plot the Δλ_peak_ signals of the small and large nanodisks as a function of time, along with the specific steps described above (Figure 6b). Comparing the Δλ_peak_ signals after the first rinse with the starting point of the experiment reveals an irreversible Δλ_peak_ of 2.2 nm for the small-disk peak and of 4.3 nm for the large-disk peak, in good agreement with results from plasmonic sensors with single-type nanodisks,^39,45,68^ thereby confirming the SLB formation. Looking then at the SiO_2_ nanosphere adsorption phase, a sudden Δλ_peak_ and distinct increase and saturation of Δλ_peak_ for both nanodisk sizes occur, corroborating the adsorption of nanospheres on the SLB. At this point, we also note that we observe different trends during the rinsing step following the exposure to POPC vesicles and SiO_2_ nanospheres in that in the former case both Δλ_peak_ signals decrease, while they increase in the latter case. Intuitively, one could assign such a peak change to a decrease and increase of the layer thickness, respectively, which, however, may not be the case, as we discuss below.

As the next step, we plot the Δλ_peak_ ratios for the entire POPC and SiO_2_ nanosphere deposition, which reproduces the key features corresponding to the different steps of the process (Figure 6c). In particular, we highlight the distinct break in overall trend at the position of the “kink”, where vesicle rupture is initiated. Next, to now be able to derive the ratio-to-thickness conversion plot, the field decay lengths of the small and large nanodisks in water are required. To obtain them, we can utilize the formed SLB, since its thickness and RI are well-known and have been quantified in multiple works to be ∼5 nm and ∼1.48, respectively.^69,70^ Here it is important to note that the formed SLB is assumed to be a nearly perfect uniform layer. While this holds for SLBs formed using POPC lipid vesicles on oxidized Si_3_N_4_, the SLB should be independently characterized in case the lipid composition, vesicle size, surface chemistry, and cleaning protocol of the substrate is changed. To this end, high-quality SLB formed on a nanodisk array similar to our case here has been confirmed.^43^ Now referring to the conversion plot, by using the thickness and RI of SLB, we can back-calculate the decay lengths of the two disks for our system in water through modification of eq 4 to
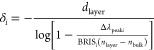
10and by assigning the thickness and RI of the SLB to the value of the Δλ_peak_ obtained during the SLB formation in our experiment: i.e., 4.3 and 2.2 nm for small and large nanodisks, respectively (Figure 6b). This procedure yields decay lengths of 14 and 29 nm for the small and large nanodisks, respectively, which are slightly different from those in air derived above, as expected.^52^ With this input, we construct the conversion plot for a water environment (Figure S6). This method of determining the decay length by using a well-known system such as an SLB is (much) less tedious than using subsequent deposition of Al_2_O_3_ layers, as we have done first. Furthermore, and most importantly, it has the additional advantage that an SLB can be efficiently removed by mild detergents after such a calibration experiment, making the nanoruler available for subsequent experiments on a system with an unknown thickness and/or RI (Figure S5).

As the final step, we use the conversion plot to extract the thickness evolution of the layers on the surface of the nanoruler during the course of our experiment (Figure 6d). Focusing again first on the SLB formation phase, we find that at the end of the rinsing step the SLB exhibits a thickness of 5 nm, which is 1 nm less than that prior to rinsing but after completed SLB formation. This can be attributed to the removal of excess lipids and lipid vesicles associated with the SLB during the rinsing and gives a first indication of the detection limit of our nanoruler. At the same time, we also highlight that the 5 nm thickness value *per se* is neither surprising nor an indication of the performance of the nanoruler, since it was used for the decay length determination above. A much more curious and significant result is observed in the phase before the SLB formation is completed: i.e., during the vesicle adsorption and rupture. Specifically, the data reveal an initial thickness buildup up to around 15 nm, after which a sudden and rapid thickness decrease occurs. Interestingly, the transition point accurately coincides with the “kink” observed in the Δλ_peak_ data, which is commonly associated with the onset of vesicle ruptures when they have reached their critical surface concentration (cf. Figure 6b). Hence, this experiment confirms the mechanism proposed to be responsible for the “kink” in a plasmonic sensor signal.^67^ Furthermore, as we elaborate later below, by establishing a correlation between the Δλ_peak_ ratio of our nanoruler and the geometry of adsorbed vesicles, we can quantitatively measure the degree of deformation of vesicles at the critical coverage before rupture.

Focusing here first on the SiO_2_ nanosphere adsorption, we see a distinct thickness increase from 5 to 16 nm, followed by a slightly reduced thickness of 14 nm after rinsing, which we attribute to the removal of loosely bound nanospheres. Since this is a cumulative thickness that includes the thickness of the SLB, we can deduce that the formed SiO_2_ nanosphere monolayer comprises particles with an average diameter of 9 nm. An independent size assessment of the nanospheres using transmission electron microscopy (TEM) reveals an average particle diameter of 11 ± 2 nm, which is in good agreement with the value derived with the nanoruler (inset in Figure 6d). We note that the diameter obtained here is slightly larger than the nominal diameter (i.e., 7 nm) and speculate that this difference arises as a consequence of the method used to determine it. To this end, the nominal diameter of the SiO_2_ nanoparticles was derived via conversion of the specific surface area (SSA) obtained using the Brunauer–Emmett–Teller (BET) method.^71^ BET, however, only permits characterization of dried samples that are prone to agglomeration and consequently results in a lower apparent SSA and thus a smaller inferred particle diameter.^72^

Last, we return to the observation that Δλ_peak_ was found to increase during the rinsing after SiO_2_ nanosphere adsorption (cf. Figure 6b), which we had tentatively ascribed to a thickness increase of the formed layer. However, as the thickness analysis reveals, this is not the case and we actually observe a slight apparent decrease in the thickness (Figure 6d), accompanied by an increase in the RI of the whole layer (Figure 6e). We speculate that the origin of these observations is the detachment of SiO_2_ nanospheres loosely attached to the dense SiO_2_ nanosphere monolayer that formed on top of the SLB. This detachment consequently lowers the overall thickness and increases the effective RI of the whole layer. We also note that a redistribution of lipids from the SLB onto SiO_2_ nanospheres would have a similar effect on the measured thickness and RI and represents an alternative explanation. Overall, the signal changes during this step are very small, and their full-scale interpretation can thus be generally debated.

To determine the resolution of the nanoruler developed here, we assess its noise in terms of both the Δλ_peak_ ratio and the deduced layer thickness at two different cases. Figure 6f,g shows the acquired Δλ_peak_ ratios at complete formation of SLB and monolayer of SiO_2_ nanospheres, respectively. Clearly, in the two cases the Δλ_peak_ ratio noises, σ_r_, are similar: that is, 0.004. This number is extremely low, and referring back to Figure 4, it is lower than the nanoruler sensitivity and therefore, considering a detection limit of 3σ_r_, it confirms the nanorulers’ ability to resolve sub 1 nm thickness changes up to an accumulated thickness of 60 nm. Indeed, this claim holds true also when we infer the resulting thickness determination noise, σ_t_, on the scale of 0.13 nm (Figure 6h,i). Therefore, with the limit of detection defined as 3σ_t_, our nanoruler should in principle be able to distinguish thickness differences of ∼0.5 nm for layers in the few tens of nanometers thickness range. This is among the highest resolutions achieved for a *functional* optical nanoruler.^22,31^

### Determination of the Degree of Vesicle Deformation prior to Rupture

As the last analysis related to the capability of our nanoruler, we investigated the dynamic changes of the response upon SLB formation in an attempt to determine the thus far elusive degree of deformation of adsorbed vesicles at the onset of SLB formation. As illustrated in Figure 6a, upon adsorption, vesicles are expected to conform to squished, truncated spheres, whose deformation at a critical elongated contact to the support triggers rupture and fusion with nearby vesicles and thus defines the onset of SLB formation. Identifying the vesicle deformation required for this onset of rupture and fusion at the critical vesicle coverage constitutes one of the most central and longstanding questions in this field.^44,47,73−75^ To this end, the related indirect data have long been indicative that the deformation required is sizable but not dramatic (see e.g. ref (76)).

**Figure 7:**
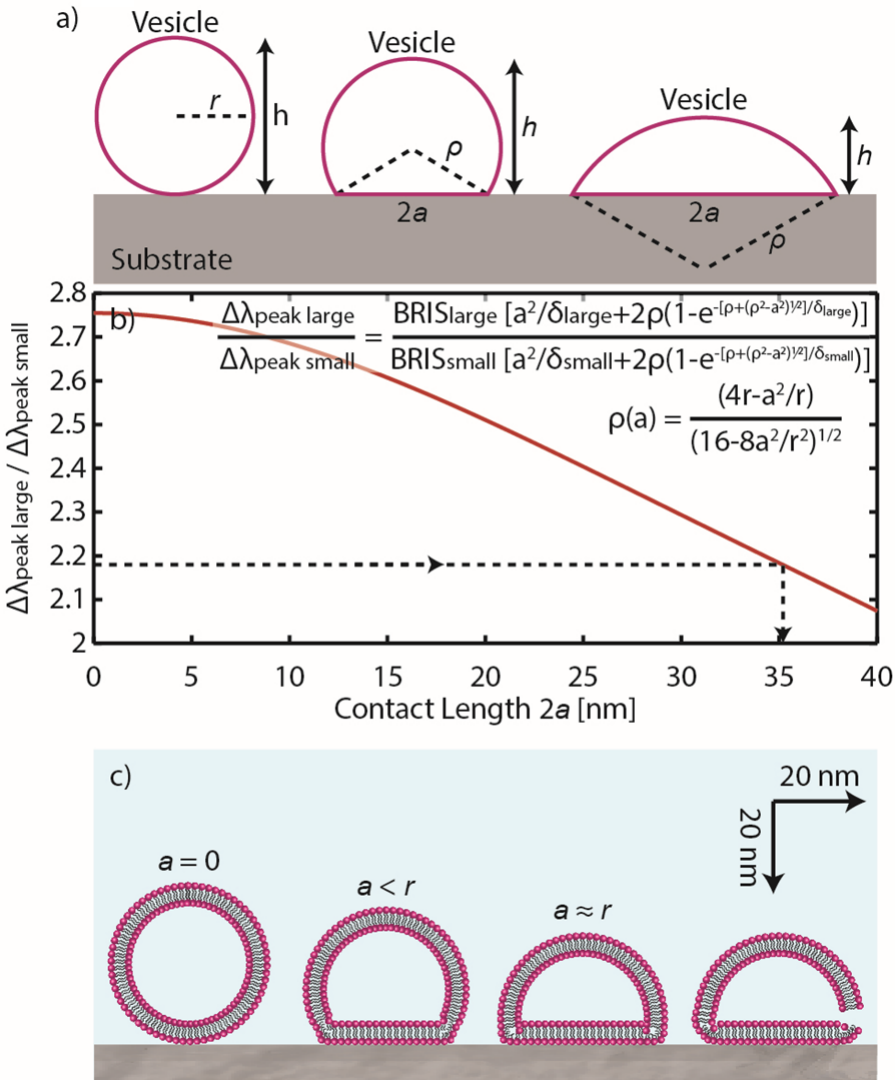
Quantifying the deformation degree of vesicles prior to rupture and SLB formation. (a) Definition of the parameters used to quantify the deformation of an adsorbed vesicle which is represented by a truncated sphere with the assumption that its surface area is preserved. *r* is the radius of an intact vesicle, *h* is the vesicle height, ρ is the radius of a deformed vesicle, and *a* is the radius of the vesicle–support contact region. (b) Nanoruler conversion plot which translates the Δλ_peak_ ratio of the large and small nanodisks (according to eq 13) into the contact length 2*a* of the deformed vesicles on top of the sensor. Translating the Δλ_peak_ ratio at the onset of rupture (i.e., Δλ_peak_ ratio of 2.18; cf. Figure 6c) to 2*a* results in a critical contact length of 35.4 nm (dashed line), which is comparable to the initial vesicle diameter. (c) To-scale schematic of the adsorption and rupture processes of the studied vesicle. The vesicle adsorbs on the support and relaxes until the contact length becomes close to its initial diameter and rupture is initiated, provided the vesicle coverage becomes equal to or is slightly above the critical coverage.

To set the stage for such determination using our nanoruler, it is crucial to recall that eq 4 operates at the level of a uniform film. In the case of adsorbed deformed vesicles, however, the observed signal should be represented as a sum of two signals corresponding to two effective films or, more specifically, to a planar region with a thickness equal to that of the SLB with a contact length defined by radius *a* and to a truncated spherical shell with the SLB thickness, radius, and height dependent on the extent of deformation (Figure 7a). In other words, eq 4 is obviously insufficient or not fully sufficient for such a case. However, as already noted, eq 4 can be extended as previously proposed for dual-band SPR^33^ or single-band LSPR.^44,47,48^ Following this line, we illustrate how the former formalism can be applied to our dual-band LSPR ruler.

To give an accurate contribution of the deformed vesicle shape to the optical signal in our sensor, we replace eq 3 by eq S1 in ref (33) (note that there is a misprint that we correct here)
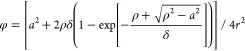
11where ρ is geometrically connected to *a* through
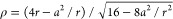
12with *r* being the initial radius of the vesicles (Figure 7a). Using eqs 1 and 11, and again converting the relevant parameters to the corresponding LSPR analogues, we express the Δλ_peak_ ratio of our nanoruler to the deformation degree of vesicles expressed via the contact length (Figure 7b)
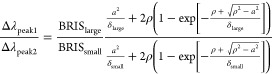
13

To finally identify the critical contact length of the deformed vesicles prior to rupture, we go back to the data presented in Figure 6c. As previously discussed, the onset of the vesicle rupture is marked by the sudden drop in the Δλ_peak_ ratio, where the ratio reaches a value of 2.18. By using the conversion plot presented in Figure 7b, the critical contact length is found to be 35.4 nm (with a corresponding height of 24.4 nm). This result is compelling, as it suggests that the vesicles will only rupture once their contact length to the support is as wide as its original diameter (Figure 7c). In a more general context, our *quantitative* result is comparable with those suggested for larger vesicles (∼100 nm) on TiO_2_ by using LSPR^44^ and for vesicles (∼170 and ∼100 nm) linked to the support via biotin–streptavidin complexes studied by using SPR^33^ and LSPR,^44^ respectively.

## Conclusions

In summary, we have developed a dual-band nanoplasmonic ruler, capable of determining in real time the change in thickness and refractive index of arbitrary (multi)layers and the shape of nanoparticles deposited on top of it, with sub-nanometer resolution. We achieved such a functionality by theoretically and experimentally devising a plasmonic sensor surface comprising two differently sized nanoantennas that independently probe adlayers and conjointly disentangling the sensor readout contribution from the adlayers’ change in thickness, refractive index, and shape. Proof-of-principle measurements in air and liquid environments corroborate the accuracy of our ruler, which is able to resolve sub-nanometer thickness changes for systems with up to 60 nm total thickness. This performance stands out among other optical nanorulers and, with its label-free, real-time, and high-throughput traits, advertises it as a promising tool to address important questions related to size and conformation in nanoscopic biological entities and, potentially, in materials science. To this end, we have here applied it to provide a direct measurement of the degree of deformation of lipid vesicles at the critical coverage just prior to rupture and SLB formation on SiO_2_. Looking forward, translating the dual-antenna concept into a flat surface type sensor^68,77^ would benefit the ruler in the form of more homogeneous evanescent fields and lack of surface corrugation. Furthermore, an even higher thickness change resolution can likely be achieved by appropriate data postprocessing.^78^

## Methods

### Sensor Fabrication

Hole-mask colloidal lithography^57^ (HCL) was used to fabricate the sensors. The details of the process undertaken (e.g., materials, fabrication steps and equipment) are described elsewhere.^52,79^ Specific to the current work, a mixture of polystyrene beads with nominal diameters of 74 and 210 nm (Interfacial Dynamics Corporation) was diluted in Milli-Q water. To ensure thorough mixing, the suspension was sonicated for at least 30 min. Specific to the HCL process, the tape-stripping step was done twice to ensure all (differently sized) polystyrene beads were completely removed. As a final step, the thin conformal Si_3_N_4_ coating layer was deposited in an STS PE-CVD system.

### Chemicals and Materials

Anhydrous chloroform (C 99%), glycerol (C 99%), HCl (1 M), NaCl (C 99%), bis(2-hydroxyethyl)aminotris(hydroxymethyl)methane (Bis-Tris, C 98%), and 1-palmitoyl-2-oleoyl-glycero-3-phosphocholine (POPC) were purchased from Merck Sigma-Aldrich (Darmstadt, Germany). The water used was of Milli-Q purity (resistivity 18.2 Ω cm, Merck Millipore, Molsheim, France). All buffers had pH = 7.0, 150 mM NaCl, and 10 mM Bis-Tris. The pH was determined using a Mettler-Toledo (Ohio, US) pH meter. Buffers were sterilized either by autoclaving at 120 °C for 20 min or by sterile filtration using 0.22 μM Stericup-GV Sterile Vacuum filters (Millipore, France). The colloidal amorphous SiO_2_ nanospheres (Bindizil 30/360; the first number denotes the weight concentration, wt %, and second number the surface area per weight, m^2^/g) were obtained from AkzoNobel PPC AB (Gothenburg, Sweden). The nominal diameter (7 nm) was calculated as the equivalent spherical diameter based on SSA measurements.

### POPC Vesicle Preparation

POPC was dissolved in chloroform and dried in a 50 mL round flask under vacuum at 60 °C using a rotavap setup. The dried lipids were left under vacuum overnight to get rid of any residual chloroform. The dried POPC was then rehydrated in H_2_O-based buffer to a concentration of 1 mg mL^–1^, followed by a very brief bath sonication to dissolve any small traces of lipids on the walls of the flask. The POPC solution underwent five freeze/thawing cycles. After which, the solution was tip-sonicated for 30 min with intervals of s 2 s pulse followed by s 3 s pause to avoid overheating of the sample. Afterward the sample was centrifuged a 20000*g* for 30 min to get rid of any residuals from the tip of the sonicator. The POPC solution was finally extruded 11 times through 30 nm polycarbonate membranes (Whatman, UK) using a mini-extruder (Avanti, USA). The resulting vesicles are typical 35 nm in diameter, as determined using dynamic light scattering (DLS).

### Flow Measurements

A commercial titanium flow cell (XNano, Insplorion AB) was used. All flow experiments were conducted under a constant flow of 100 μL/min, as regulated by a peristaltic pump (Ismatec). The sensor was illuminated using a fiber-coupled halogen lamp (AvaLight-Hal, Avantes), while the extinction spectra were continuously recorded by a fiber-coupled fixed grating spectrometer (AvaSpec-HS-TEC, Avantes). Bulk refractive index sensitivity was derived by exposing the nanoruler surface to mixtures of Milli-Q water (Millipore) and ethylene glycol (Sigma-Aldrich) at the mixing ratios 100:0, 80:20, 60:40, 40:60, and 20:80 wt %. The λ_peak_ response was derived by fitting a Lorenztian to the spectra.^79^ Prior to flow experiments, the sensor was exposed to UV/ozone for 3 min. This UV/ozone treatment transformed the Si_3_N_4_ coating surface into SiO_2_, with the SiO_2_ thickness depending on the O_3_ partial pressure, UV irradiance, and duration of exposure of the surface.^80^

### Decay Length Determination Using Al_2_O_3_ Layer-by-Layer Deposition

To determine the decay lengths of the two nanodisk populations on the nanoruler surface, subsequent thin Al_2_O_3_ layers were grown by atomic layer deposition (ALD, Oxford FlexAl). Intermittently, the layer thickness (deposited on an analogous silicon chip simultaneously) was evaluated by ellipsometry (J.A. Woollam M2000) and the extinction spectra were recorded using a Cary 5000 spectrophotometer. Great care was taken to ensure that the spectra were always acquired from the same spot on the sample.

### TEM Measurements

To enable imaging by TEM, the colloidal silica particles were deposited on commercial electron-transparent substrates consisting of a holey-carbon film on a copper grid. The particles were imaged using a FEI Tecnai T20 microscope, equipped with a LaB_6_ filament and operated at 200 kV.

### FDTD Simulations

The electromagnetic simulations were carried out using the finite-difference time-domain method as implemented in the software FDTD Solutions. The Au nanodisks, whose permittivity was taken from measurements by Johnson and Christy,^81^ were modeled as flat cylinders with both top and bottom edges being rounded. The radius and height of the nanodisks spanned the range from 20 to 90 nm and from 20 to 70 nm, respectively. The nanodisks were placed on a substrate with RI = 1.5. When modeling bulk sensitivity, the superstrate’s RI was varied from 1.33 to 1.5. For local sensing of a conformal layer, the superstrate was taken always as water (*n* = 1.33). The thickness of the conformal layer, which covered both the nanodisk and the substrate, ranged from 1 to 20 nm and its RI spanned from 1.37 up to 1.5. The simulation volume around the LSPR sensor had a mesh step of 0.5 nm. Perfectly matched layer absorbing boundary conditions were used to terminate the simulation volume, and a linearly polarized plane wave excitation source was introduced via a total-field/scattered-field scheme.

## Notes

A preprint version of this work is published elsewhere.^82^

## Supporting Information Available

Bulk refractive index sensitivity–geometry correlation in Au nanodiskx, physical and sensitivity characterizations of the dual-peak nanorulers, and construction of Δλ_peak_ ratio–layer thickness in a single experiment in liquid medium (PDF)
