# Supplementary material for: Time-Resolved Thickness and Shape-Change Quantification using a Dual-Band Nanoplasmonic Ruler with Sub-Nanometer Resolution
Source: ACS Nano. 2022 Sep 9;16(10):15814–26. doi: 10.1021/acsnano.2c04948 (PMC9620406; doi:10.1021/acsnano.2c04948)
Supplement: Supplementary file 1 — nn2c04948_si_001.pdf [file nn2c04948_si_001.pdf]

Supporting Information

Time-Resolved Thickness and Shape-Change Quantification using a Dual-Band Nanoplasmonic Ruler with Sub-Nanometer Resolution

Ferry Anggoro Ardy Nugroho[†,‡,§,*], *Dominika Świtlik*[^], *Antonius Armanious*[‖], *Padraic O'Reilly*[†], *Iwan Darmadi*[†], *Sara Nilsson*[†], *Vladimir P. Zhdanov*[†,∇], *Fredrik Höök*[†], *Tomasz J. Antosiewicz*[†,^,*] *and Christoph Langhammer*[†,*]

[†]Department of Physics, Chalmers University of Technology, 412 96 Göteborg, Sweden

[‡]Department of Physics and Astronomy, Vrije Universiteit Amsterdam, De Boelelaan 1081, 1081 HV Amsterdam, The Netherlands

[§]Department of Physics, Universitas Indonesia, Depok 16424, Indonesia

[^]Faculty of Physics, University of Warsaw, Pasteura 5, 02-093, Warsaw, Poland

[‖]Department of Health Sciences and Technology, ETH Zurich, 8092 Zurich, Switzerland

[∇]Boreskov Institute of Catalysis, Russian Academy of Sciences, Novosibirsk 630090, Russia

Correspondence to: ferryanggoroardynugroho@yahoo.com; tomasz.antosiewicz@fuw.edu.pl; clangham@chalmers.se

1. **Bulk Refractive Index Sensitivity-Geometry Correlation in Au Nanodisk**

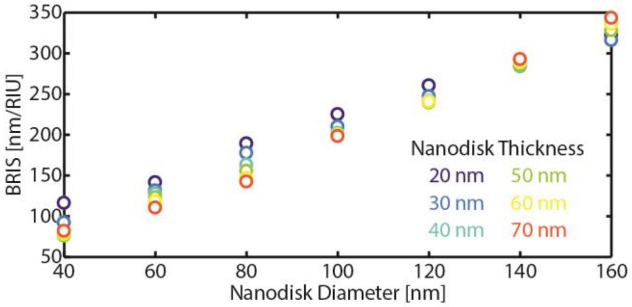

Supplementary Figure 1. *FDTD-calculated bulk refractive index sensitivity (BRIS) of a Au nanodisk with different dimensions. Quasi-linear dependence of the BRIS on the nanodisk diameter is observed.*

2. **Physical and Sensitivity Characterizations of the Dual-Peak Nanorulers**

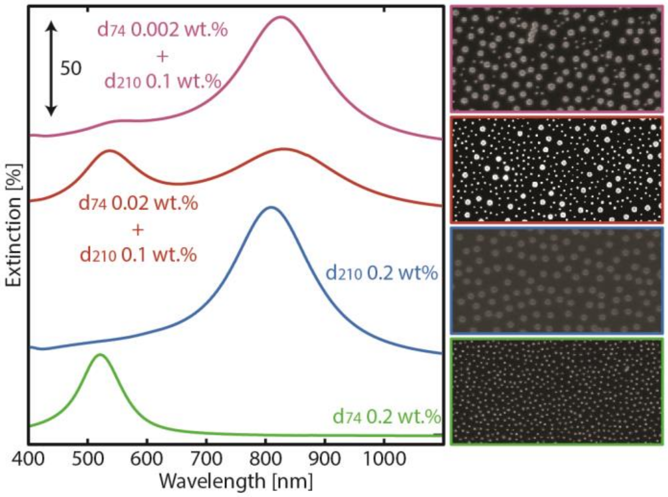

Supplementary Figure 2. *Left: Optical extinction spectra of nanorulers with different compositions of the Ag nanodisk array, as determined by the mixing ratio of the mixed polystyrene bead suspension used during nanofabrication. Clearly, the relative intensity of the extinction peak corresponding to the respective Ag nanodisk diameter is controlled by the mixing ratio of the polystyrene beads in the suspension. Right: Scanning electron microscope (SEM) images of the corresponding nanoruler surfaces. The panels are 2×4 μm²*.

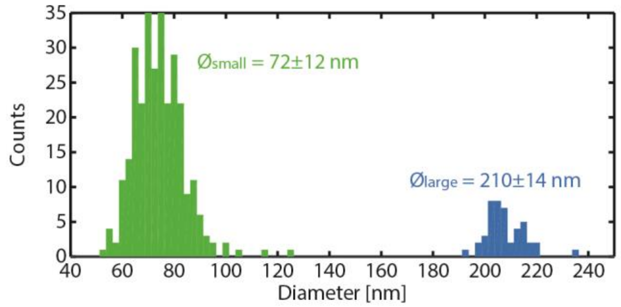

Supplementary Figure 3. *Small and large nanodisk diameter distribution histograms derived from SEM images.*

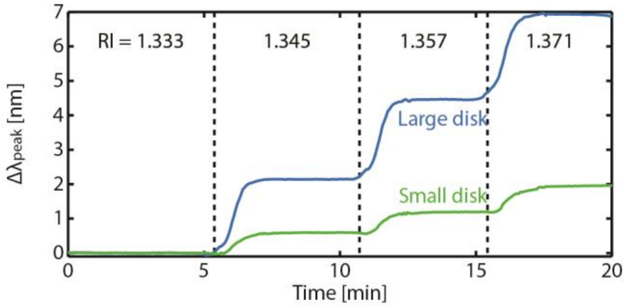

Supplementary Figure 4. *Time-resolved $\Delta\lambda_{peak}$ response of the small and large nanodisks upon exposure to water:glycerol mixtures with different composition and thus different refractive indices.*

3. Constructing $\Delta\lambda_{peak}$ Ratio-Layer Thickness in a Single Experiment in Liquid Medium

When utilizing the nanoruler in the flow-mode or experiments in liquid medium, there is one clear advantage compared to the ones carried out in air, in that both sensitivity characteristics of the sensors (*BRIS* and decay lengths) can be determined one after the other, *prior to* the actual measurements. This possibility does not only simplify the experiment steps but also ensures that the sensitivity characteristics are derived from the *exact* sensor used, thus warranting correct $\Delta\lambda_{peak}$ ratio-layer thickness conversion plot employed (in contrast to, for example, measuring these on one sensor and then assuming they are valid for other sensors fabricated in the same batch).

The steps to do this are illustrated in **Supplementary Figure 5**. One can start with measuring the *BRIS* by exposing the sensor to solutions with different refractive indices. Upon flushing back with the original solution (herein we used buffer solution), the sensor reverts to its original condition and thus one can move on to the second step: measuring the decay lengths. As discussed in the main text, we can measure decay lengths efficiently using the POPC SLB formed on top of the sensor, so that its characteristics are well-known (d_{layer} = 5 nm, n_{layer} = 1.48).[1,2] As detailed in **Supplementary Figure 5**, using these two parameters we can then infer the decay lengths of each of the nanodisks in the sensor, and subsequently construct the $\Delta\lambda_{peak}$ ratio-layer thickness conversion plot. The advantageous aspect of using POPC to derive the decay lengths is that it can be efficiently removed afterwards by using sodium dodecyl sulfate (SDS). This is apparent as shown in **Supplementary Figure 5**, where the $\Delta\lambda_{peak}$ shifts back to 0 nm after such "cleaning", indicating a sensor surface with similar state as the one at the beginning. From this point one can then proceed with the desired experiment.

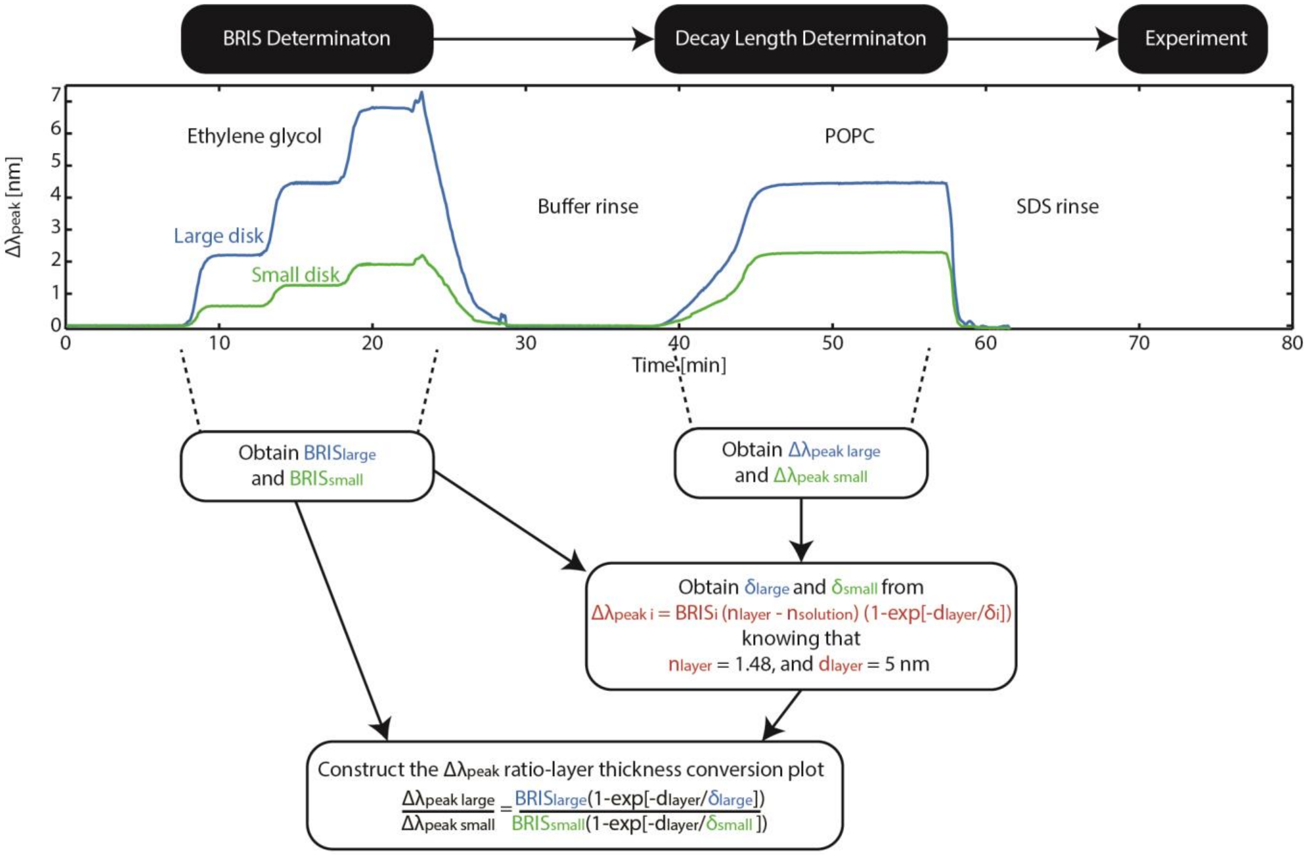

Supplementary Figure 5. *Sequence of experiment steps executed to construct the $\Delta\lambda_{peak}$ ratio-layer thickness plot from a single experiment.*

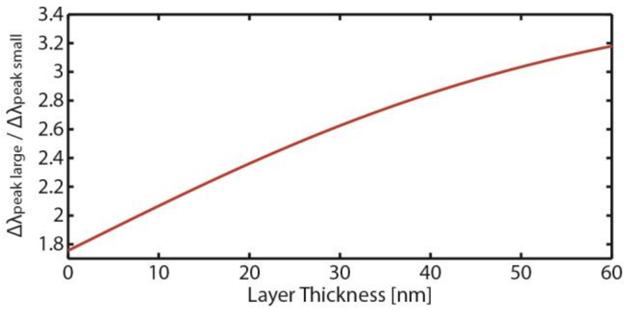

Supplementary Figure 6. *Nanoruler $\Delta\lambda_{peak}$ ratio-layer thickness conversion plot in water, derived from the POPC SLB measurement. See main text for details.*

REFERENCES

(1) Dave, P. C.; Tiburu, E. K.; Damodaran, K.; Lorigan, G. A. Investigating Structural Changes in the Lipid Bilayer upon Insertion of the Transmembrane Domain of the Membrane-Bound Protein Phospholamban Utilizing 31P and 2H Solid-State NMR Spectroscopy. *Biophys. J.* **2004**, *86* (3), 1564–1573. https://doi.org/10.1016/S0006-3495(04)74224-1.

(2) Huber, T.; Rajamoorthi, K.; Kurze, V. F.; Beyer, K.; Brown, M. F. Structure of Docosahexaenoic Acid-Containing Phospholipid Bilayers as Studied by 2H NMR and Molecular Dynamics Simulations. *J. Am. Chem. Soc.* **2002**, *124* (2), 298–309. https://doi.org/10.1021/ja011383j.
